# Joubert Syndrome Presenting with Motor Delay and Oculomotor Apraxia

**DOI:** 10.1155/2011/262641

**Published:** 2012-01-26

**Authors:** Harjinder Gill, Brinda Muthusamy, Denize Atan, Cathy Williams, Matthew Ellis

**Affiliations:** ^1^Community Paediatrics, The Children's Hospital, Oxford University Hospitals, Headley Way, Headington, Oxford OX3 9DU, UK; ^2^Paediatric Ophthalmology and Adult Strabismus, The Wilmer Eye Institute at Johns Hopkins, 600 North Wolfe Street, Baltimore, MD 21287, USA; ^3^Academic Department of Ophthalmology, School of Clinical Sciences, Bristol Eye Hospital, Lower Maudlin Street, Bristol BS1 2LX, UK; ^4^Centre for Child and Adolescent Health, School of Social and Community Medicine, University of Bristol, Oakfield House, Oakfield Grove, Bristol BS8 2BN, UK

## Abstract

We describe two sisters who presented in early childhood with motor delay and unusual eye movements. Both demonstrated hypotonia and poor visual attention. The older girl at 14 weeks of age showed fine pendular horizontal nystagmus more pronounced on lateral gaze, but despite investigation with cranial MRI no diagnosis was reached. The birth of her younger sister four years later with a similar presentation triggered review of the sisters' visual behaviour. Each had developed an unusual but similar form of oculomotor apraxia (OMA) with head thrusts to *maintain* fixation rather than to *change* fixation. MRI of the older sibling demonstrated the characteristic “molar tooth sign” (MTS) of Joubert syndrome which was subsequently confirmed on MRI in the younger sibling. We discuss the genetically heterogeneous ciliopathies now grouped as Joubert syndrome and Related Disorders. Clinicians need to consider this group of disorders when faced with unusual eye movements in the developmentally delayed child.

## 1. Introduction

Joubert syndrome (JS) is a rare genetic disorder first described in 1968 [1]. It is characterised by developmental delay, hypotonia, and ataxia with the pathognomonic finding of a “molar tooth sign” (MTS) on MRI imaging of the brain. This refers to the abnormal structural features of cerebellar vermis hypoplasia, deepened interpeduncular fossa, and elongated, horizontally orientated thickened superior cerebellar peduncles. The term Joubert syndrome and related disorders (JSRDs) describes conditions that share the MTS and the clinical features of Joubert syndrome but that also have other clinical manifestations involving the CNS (occipital encephalocele, corpus callosal agenesis), eyes (coloboma, retinal dystrophy, nystagmus, oculomotor apraxia), kidneys (nephronophthisis, cystic dysplasia), liver (hepatic fibrosis), and limbs (polydactyly).

Here, we describe 2 sisters that presented with developmental delay and eye movement abnormalities. Subsequently, they were found to have the pathognomonic MTS on MRI brain imaging. The clinical sign that finally led to the diagnosis was the unusual character of the eye movement disorder (oculomotor apraxia) exhibited by both siblings. The family have given their consent for the publication of this paper.

## 2. Case Report

AZ was the first child to nonconsanguineous parents born weighing 3.26 kg at term in good condition (Apgars 8^1^, 9^5^, 10^10^) by spontaneous vaginal delivery following an uneventful pregnancy and delivery. At 20 hours postpartum she was reassessed due to respiratory distress. Chest X-ray suggested probable transient tachypnoea of the newborn, and she was managed conservatively. On day 3 she developed vomiting after feeds and apnoeic episodes. A full septic screen was normal. An oesophageal pH study showed moderate reflux. She was discharged home on day 11 with antireflux medications. 

By 10 weeks the family noted AZ to be having difficulty visually fixing on smiling faces. Medical review confirmed poor visual attention with both eyes tending to deviate to one side the majority of the time. In addition she tended to hold her mouth open with a prominent protruding tongue with soft neurological signs of mild head lag and some fisting of the hands. As a result ophthalmic review and head MRI was arranged.

At 14 weeks the paediatric ophthalmologist noted delayed but improving visual attention, normal ocular examination, and a fine pendular horizontal nystagmus more pronounced on lateral gaze. An MRI at this time was reported to be normal although in retrospect the superior cerebellar peduncles appeared thicker than normal.

At 8 months, AZ was demonstrating evidence of gross motor developmental delay: she was not yet rolling over and tended to thrust herself backwards from a sitting position. On examination she was hypotonic with unusual tongue thrusting movements. A neurogenetic opinion was sought. Differential diagnoses considered at this time were a possible neuromuscular condition, Angelman or Prader Willi. Relevant genetic investigations were normal. At subsequent review at 30 months, AZ was functioning at an approximately 12–18-month level in all skill areas.

Shortly after her sister, BZ was born weighing 3.72 Kg at term with Apgars of 5^1^, 9^5^, 10^10^ following an uneventful pregnancy and delivery. Her neonatal course was uneventful. At 2 months of age, her family reported her breathing to be noisy, but no abnormality was detected on examination.

At review at 7 months of age BZ was found to be delayed in her motor development with hypotonia and similar visual tracking problems to her sister. Paediatric ophthalmology review now confirmed that both sisters had an unusual form of oculomotor apraxia. AZ, now age 4, had disordered eye movements with unusual head thrusts—in effect she moved her head to *maintain* fixation rather than to *change* fixation. BZ at age two demonstrated similar abnormalities. 

**Figure 1:**
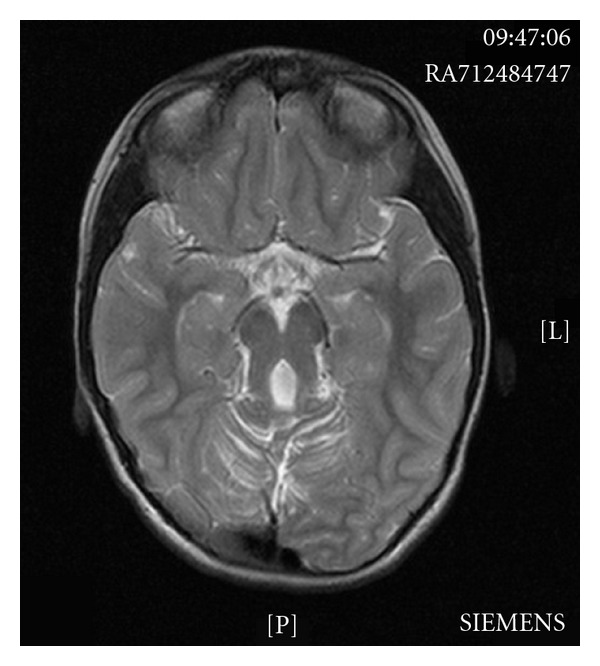
Cranial T2 magnetic resonance image for AZ—this shows a midline defect in the cerebellar vermis, thickened horizontally orientated cerebellar peduncles, a small midbrain, and dysplasia of the cerebellar cortex. This demonstrates the characteristic “molar tooth sign” seen in Joubert Syndrome and JSRD.

**Figure 2:**
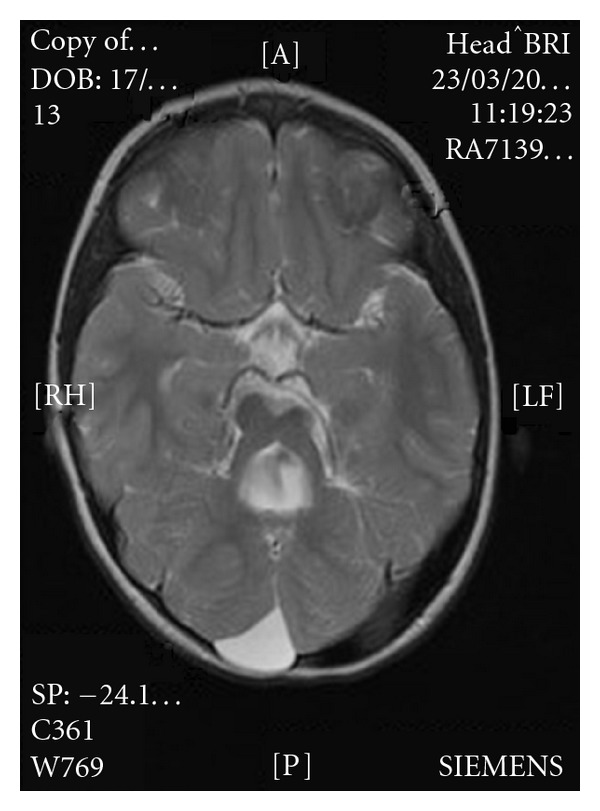
Cranial T2 magnetic resonance image for BZ—this shows an enlarged 4th ventricle with cerebellar vermis hypoplasia and horizontally orientated superior peduncles. There is also a posterior fossa arachnoid cyst.

A repeat MRI was organised for AZ to look in detail at the cerebellar region. This demonstrated a midline defect in the cerebellar vermis, thickened horizontally orientated cerebellar peduncles, and a small midbrain (Figure 1). Consequently BZ also underwent MRI imaging which demonstrated an enlarged 4th ventricle with cerebellar vermis hypoplasia and horizontally orientated superior cerebellar peduncles (Figure 2). These features are consistent with the “molar tooth sign” that is pathognomonic of Joubert syndrome. BZ also has a posterior fossa arachnoid cyst which is one of the CNS malformations also associated with JSRD. The family have declined further genetic testing looking for specific mutations as they do not feel it will add further to their diagnosis and subsequent management. 

## 3. Discussion

The incidence of JSRD has been estimated to be between 1/80,000 and 1/100,000 live births [2–4]. Joubert syndrome and related disorders are genetically heterogenous with mutations in 13 genes known to cause Joubert syndrome to date (OMIM). Most demonstrate autosomal recessive inheritance, although cases of X-linked inheritance (CXORF5) and autosomal dominant inheritance (TTC21B) have been described (Table 1).

Known causative genes encode proteins that localise to the primary cilium; therefore, classifying Joubert syndrome as one of a number of so-called “ciliopathies”. The primary cilium is a nonmotile type of cilium that is found on nearly every cell of the body [5]. Previously thought to be a nonfunctional vestigial organelle, the primary cilium is now known to act like a scaffold for several signalling pathways, such as sonic hedgehog (Shh), Wnt, and platelet-derived growth factor (PDGF*α*) that are critically important for normal cell development and differentiation. Furthermore, the ciliopathies demonstrate several overlapping clinical features, such as retinal dystrophy and renal disease. The relationship between these disorders is clearly demonstrated by the allelic heterogeneity of common causative genes. For example, different mutations of the JBTS5 gene, CEP290, are known to cause ciliopathies that predominantly affect the retina, Bardet-Biedl syndrome and Leber's congenital amaurosis, as well as Joubert's syndrome and related disorders, Senior-Løken and Meckel's syndromes [6] (Table 1). Furthermore, intrafamilial phenotypic heterogeneity suggests the existence of multiple genetic modifiers [7]. Consequently, adequately predicting the underlying mutation in families with JSRD can be difficult and only possible in 50% of cases [8]. Nevertheless, emerging phenotype-genotype correlations may help in this regard (Table 1). Clearly, many different ophthalmic manifestations can be associated with JSRD (see below), and so the identification of the characteristic OMA that we describe in 2 siblings may be indicative of a specific genetic defect.

## 4. Oculomotor Abnormalities in JSRD 

Abnormal eye movements are a frequent finding in JSRD. These are independent of any specific ocular features seen in the subtypes and are consistent with structural malformation of the neural pathways [10]. Clinical observation of the oculomotor signs suggests that there is disruption in the pathways linking the superior colliculus and upper midbrain with the paramedian pontine reticular formation and medial longitudinal fasciculus. 

Oculomotor apraxia is an impairment of planning and organization of voluntary conjugate movements of the eyes. Saccadic initiation failure is the commonest eye movement abnormality in JSRD [11] (saccades: when the eyes rapidly change fixation from one target to another). In JSRD, the saccades are either hypometric (reduced amplitude) or absent. Both the horizontal and vertical initiation of saccadic eye movements are affected. Contrast this to congenital oculomotor apraxia which primarily affects horizontal saccades [12]. To compensate for the absence of saccadic eye movements, the patient learns to use a thrusting motion of the head to change fixation. This is sometimes preceded by a blink to break fixation when moving from one target to another. 

Smooth pursuit of a target in motion is typically slow in JSRD, and patients produce a “catch-up” saccade or head thrust, to maintain fixation. Poor cancellation of the vestibulo-ocular reflex is also observed [10, 12]. These findings correlate with hypoplasia of the posterior cerebellar vermis. 

Pendular, rotary, horizontal, vertical, and see-saw nystagmus are all described in JSRD depending on the extent of cerebellar and midbrain involvement [11–13]. Pendular nystagmus appears to be associated more with severe visual impairment, retinal pigmentary changes, and attenuated electroretinogram measurements [11, 12]. Hodgkins et al. describe a unique finding of “periodic horizontal alternating gaze shifts” where the patient shifts gaze every 5 to 15 seconds between extreme horizontal gaze positions. They feel that this is a form of periodic alternating nystagmus without the quick phase due to the failure to initiate saccades. 

Strabismus is not uncommon in JSRD and can present as either horizontal or vertical misalignment. Horizontal strabismus can be an exodeviation, esodeviation, fixed or alternating in nature. 

Findings on retinal examination include drusen of the optic nerves and mottled pigmentation of the peripheral retina. Retinal pigmentation is found to be associated with poorer visual acuity and abnormalities of the renal system [11, 12]. 

## 5. Conclusion 

The specific learning point from these 2 sisters was the significance of the neurological impairments in combination with the abnormal eye movements leading to the final diagnosis. We recommend careful analysis of the cerebellar region on MRI in a child who has the neurological features of hypotonia, ataxia, and developmental delay with eye movement abnormalities or oculomotor apraxia (OMA), specifically looking for the “molar tooth sign” and vermis hypoplasia of the cerebellum. In families affected by JSRDs, genotype-phenotype correlation may improve our understanding of this fascinating group of developmental impairments in the future. 

## Figures and Tables

**Table 1:** Molecular genetics of Joubert's syndrome (adapted from Parisi) [7].

	Gene symbol	Locus and inheritance		Protein name	Localisation	MTS	Liver	Col	RD	Renal	Poly	OE	OMA	Associations with other ciliopathies
JBTS1	INPP5E	9q34	Ar	Inositol polyphosphate-5-phosphatase E	BB, PC	++	−	−	+	−	−	−	+	—
JBTS2	TMEM216	11q13	Ar	Transmembrane protein 216	BB, PC	++	−	+	+	+	+	+	++	MKS
JBTS3	AHI1	6q23	Ar	Jouberin/Abelson helper integration site 1	BB, PC	++	−	+/−	++	+	−	−	+	NPHP
JBTS4	NPHP1	2q13	Ar	Nephrocystin-1	BB, PC	+/−	−	−	+	++	−	−	+	SLSN, NPHP
JBTS5	CEP290	12q21	Ar	Centrosomal protein of 290 kDa	BB, PC	++	+	+	++	++	−	+	+/−	MKS, LCA, BBS, SLSN
JBTS6	TMEM67	8q21	Ar	Transmembrane protein 67/Meckelin	BB, PC	++	++	+	−	+	+/−	+	+/−	COACH, MKS, NPHP, BBS (modifier)
JBTS7	RPGRIP1L	16q12	Ar	RPGR-interacting protein 1-like protein	BB, PC	++	+	+/−	+/−	++	+	+	+/−	COACH, MKS
JBTS8	ARL13B	3q11	Ar	ADP-ribosylation factor-like 13B	BB, PC	++	−	−	+	−	−	+	−	—
JBTS9	CC2D2A	4p15	Ar	Coiled-coil and C2 domains-containing protein 2A	BB	++	+	+	+	+	−	+	+/−	COACH, MKS
JBTS10	CXORF5	Xp22	Xr	Chromosome X open reading frame 5	PC	+	−	−	+	−	+	−	−	OFD1, SGBS2
JBTS11*	TTC21B	2q24	Ad	Tetratricopeptide repeat-domain 21B	IFT, PC	+	?	?	?	?	?	?	?	ATD4, NPHP
JBTS12	KIF7	15q26	Ar	Kinesin family member 7	IFT	+	−	−	−	−	+	−	−	ACLS, HLS2
JBTS13	TCTN1	12q24	Ar	Tectonic 1	BB, PC	+	−	−	−	−	−	−	−	—

*The paper describing JBTS11 by Davis et al. [9] screened for TTC21B mutations in patients with classic Joubert's syndrome but does not mention associated clinical features.

Ar: autosomal recessive; Ad: autosomal dominant; Xr: X-linked recessive; BB: basal body; PC: primary cilium; IFT: intraflagellar transport; MTS: molar tooth sign; Col: coloboma; RD: retinal dystrophy; Poly: polydactyly; OE: occipital encephalocele; OMA: oculomotor apraxia; SLSN: Senior-Løken syndrome; BBS: Bardet-Biedl syndrome; LCA: Leber's congenital amaurosis; COACH: Coloboma, Oligophrenia/developmental delay, Ataxia, Cerebellar vermis hypoplasia, Hepatic fibrosis; MKS: Meckel syndrome; NPHP: Nephronophthisis; OFD1: oral-facial-digital syndrome 1, SGBS2: Simpson-Golabi-Behmel syndrome type 2; ATD4: Asphyxiating thoracic dystrophy type 4; ACLS: Acrocallosal syndrome; HLS2: Hydrolethalus syndrome 2.
